# An objective assessment method for frequency selectivity of the human auditory system

**DOI:** 10.1186/1475-925X-13-171

**Published:** 2014-12-18

**Authors:** Qin Gong, Yao Wang, Meng Xian

**Affiliations:** Department of Biomedical Engineering, School of Medicine, Tsinghua University, Beijing, 100084 China; Research Center for Biomedical Engineering, Graduate School at Shenzhen, Tsinghua University, Shenzhen, 518055 China

**Keywords:** Stimulus frequency otoacoustic emission suppression tuning curves (SFOAE STCs), Psychophysical tuning curves (PTCs), Frequency selectivity (FS) assessment

## Abstract

**Background:**

Frequency selectivity (FS) is an important aspect of auditory function, and is typically described by a tuning curve function. Sharply tuned curves represent a higher acuity in detecting frequency differences, and conversely, broadly tuned curves demonstrate a lower acuity. One way of obtaining tuning curves is from techniques based on subjective behavioral responses, which yields psychophysical tuning curves (PTCs). In contrast, other methods rely on objective auditory responses to sound, such as neuron responses and otoacoustic emissions, amongst others. The present study introduces an objective method that uses stimulus frequency otoacoustic emissions (SFOAEs) to assemble suppression tuning curves (STCs). Finding an objective method of accurately measuring human FS is very important, as it would permit the FS to be assayed in non-responsive patients (e.g., neonates or comatose patients). However, before the objective method can be applied, it must be demonstrated that its ability to estimate the FS, gives comparable results to those obtained by subjective procedures i.e. PTCs.

**Methods:**

SFOAEs responses, generated in the peripheral auditory system, were used to produce STCs. PTCs were measured by behavioral responses. The validity of the objective measures of human FS were determined by comparing stimulus frequency otoacoustic emission suppression tuning curves (SFOAE STCs) to PTCs at common stimulus parameters in 10 individuals with normal hearing, at low probe-tone levels.

**Results:**

The average Q_10_ ratios measured between PTCs and SFOAE STCs from subjects were close to 1 at various center frequencies (*F*_2,24_ = .15, *p* = .858). The estimates of FS provided by SFOAE STCs and PTCs were similar.

**Conclusions:**

This system could be used to estimate auditory FS by both objective and subjective methods. SFOAE STCs have the potential to provide an objective estimate of auditory FS.

## Background

Frequency selectivity (FS) refers to the ability of the auditory system to identify tonal components in complex sound [1]. It largely depends on the filtering ability of the cochlea [2], and its tuning properties are determined by the amplification mechanisms of the cochlear outer hair cells (OHCs) at low stimulus levels [3–5]. Damage to the OHCs will reduce both the FS and the sensitivity of the auditory system [6–11], with far-reaching consequences for complex sound perception [12]. The reduction or abolition of other OHC-related phenomena, such as two-tone suppression [13, 14] or otoacoustic emissions [15], will also damage cochlear FS. Consequently, the estimation of FS can indirectly assess OHC function and has a significant effect on complex sound perception.

The evaluation of FS is actually a measurement of the bandwidth of the auditory filter on the basilar membrane. Fletcher [16] measured the threshold of a sinusoidal signal as a function of the bandwidth of a band-pass noise masker, referring to the bandwidth of the auditory filter as the “critical bandwidth”, a term that was adopted in later studies [17–20]. Auditory masking can be used to estimate auditory filter shapes [21]. Psychophysical tuning curves (PTCs) can assess FS by using the psychoacoustic detection of masked signals to obtain tuning curves. Since a behavioral response of sound perception is required from the subject, this is considered a subjective hearing evaluation. In humans, forward masking PTCs are more sharply tuned than simultaneous masking PTCs [22], probably because forward masking PTCs overestimate the sharpness of the frequency tuning [23]. The traditional method of obtaining PTCs is time-consuming, because it requires a series of stimulus generation and feedback steps to find the masker intensity at each frequency. However, in 2005, Sek *et al.* developed a faster method for determining PTCs by using a narrowband noise filter with a center frequency being swept from low to high frequencies [24]. This method only takes 8 minutes to perform. Despite this time-efficient way of obtaining PTCs, their interpretation is influenced by non-auditory factors such as attention [25], therefore it cannot be used for difficult-to-test populations (e.g., age < 3 years, especially neonates).

Otoacoustic emissions (OAEs) are weak acoustic signals produced in the cochlea. They arise from non-linear inner ear mechanics and are detected as acoustic signals in the ear canal [26], making them a useful noninvasive measurement. Stimulus frequency otoacoustic emissions (SFOAEs) are evoked OAEs that have the same frequency as the stimulus. SFOAEs can be evoked within a wide frequency range for subjects with normal hearing or with moderate hearing impairment. The SFOAE suppression tuning curves (SFOAE STCs) are detected by using a suppression-based mode similar to simultaneous masking. Kemp and Chum [27] found that a subject’s SFOAE STCs and PTCs showed a similar filter shape, suggesting that SFOAE STCs could potentially be used to evaluate the periphery auditory system objectively. However, this possibility has not been sufficiently explored in the literature. Siegel *et al*. [28] obtained the SFOAE STCs of a chinchilla at a stimulus frequency of 9 kHz, which were similar to the STCs constructed from the suppressed discharge patterns of the auditory nerve fibers obtained by Temchin, Rich & Ruggero [29]. Keefe *et al.* [30] predicted that the two-tone suppression of SFOAEs in the human ear would resemble the results of a simultaneous masking behavioral test. Cheatham *et al.* [31] found that the tuning characteristics of SFOAEs provided signal processing information prior to inner hair cell stimulation and auditory nervous activation. Charaziak *et al.* [32] compared the average SFOAE STCs and PTCs in 10 normal-hearing subjects for a probe frequency centered around 1,000 and 4,000 Hz, at low probe levels. They concluded that SFOAE STCs are useful for estimating behavioral tuning noninvasively at the group level, but not at the individual level because of the variability in individual SFOAE STCs.

In the aforementioned studies, the relationships of factors such as Q_10_ ratio and tip offset were not quantified between PTCs and SFOAE STCs. In the present study, we sought to develop an assessment system for human auditory FS containing both objective detection of the SFOAE STCs and subjective detection of the simultaneous masking PTCs. We investigated the relationships between the two tests, at low probe level (30 dB SPL) in 10 normal hearing subjects, based on the statistical analysis of the tuning curve parameters. Similarities were found between the estimates of FS provided by the SFOAE STCs and the PTCs.

## Methods

### Subjects

Ten subjects (20–26 years old, 6 females, 4 males) were included in the study, all of whom were native Chinese speakers and college students at Tsinghua University. In accordance with the inclusion criteria, all participants had normal otoscopic examination results, normal hearing thresholds (<15 dB HL for octave frequencies of 250–8,000 Hz), no spontaneous OAEs (SOAEs) in the frequency range of interest (to avoid interference with the detection signal), and no history of neurological or psychiatric illness. All subjects gave their written informed consent to participate, in compliance with a protocol (IRB00008273) approved by the institutional review board at Tsinghua University.

### System set-up

All experiments were conducted in an acoustic booth. Stimulus generation, signal acquisition, and processing were performed through an external soundcard (Fire face 800, RME, Haimhausen, German, 24-bit resolution, 192 kHz sampling rate) controlled by a Windows-based computer system. An audio stream input/output (ASIO, Steinberg, Hamburg, German) is a computer soundcard driver protocol for digital audio, allowed us to access external hardware directly, without using Microsoft’s DirectSound. It was used to provide a low-latency and high-fidelity interface between the assessment system and soundcard. A probe, containing miniature loudspeakers and a microphone, was inserted into the subject’s ear. The external soundcard converted a computer-generated digital signal to an analog voltage signal, which was transduced to an acoustic signal by the loudspeakers (ER-2, Etymotic Research, London, USA) and delivered to the ear via tubes. A miniature microphone (ER-10B+, Etymotic Research, London, USA) transduced the acoustic signal collected in the ear canal to an analog voltage signal, which was amplified by 20 dB (ER-10B+ preamplifier, Etymotic Research, London, USA), converted to a digital signal via the external soundcard, and sent back to the main control computer. In the detection of PTCs, the subject indicated the detection of a probe tone by pressing a USB handle button. The assessment system was based on the C sharp programming language (Microsoft Inc., Redmond, Washington, USA) and embedded within Matlab (MathWorks Inc., Natick, MA, USA) for cross-programming. Analysis of variance (ANOVA) and paired-sample *t* tests were conducted in SPSS (SPSS Inc., Chicago, IL, USA).

### Calibration

Calibration was conducted in a Brüel & Kiær ear simulator (type 4157) at the frequencies of 125, 250, 500, 1000, 2000, 3000, 4000, 6000 and 8000 Hz, for both tone and narrowband noise. At each frequency and intensity, six input–output (I/O) signals were recorded, including the digital output generated from the computer, the analog output delivered from soundcard, the sound pressure in the ear simulator, the sound pressure collected in the miniature microphone, the analog input collected by soundcard and the digital input gathered by computer. The I/O functions of the system were calculated by interpolation or extrapolation of the calibration data. The sound pressure level (SPL) measured in decibels (dB), was referenced to 20 μPa.

### Experimental procedure

**Figure 1:**
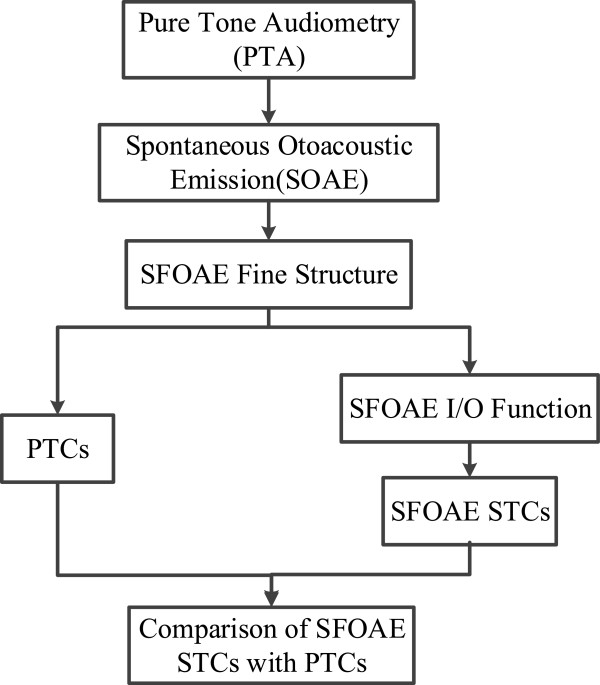
**Flowchart of the experimental procedure.** A flowchart of the experimental procedure.

A flowchart of the experimental procedure is shown in Figure 1. Pure tone audiometry (PTA) and SOAEs tests were examined to select eligible subjects. SFOAE fine structure was a high-resolution (40-Hz steps) SFOAE recording in the frequency range of ±200 Hz (relative to the center frequency, CF) to define the best frequency of the probe for testing the SFOAE STCs and PTCs. The best frequency, denoted as *f*_*p*_ in the subsequent tests, is the one that can evoke the largest SFOAE. The probe level, *L*_*p*_, was 30 dB SPL. The suppressor frequency, *f*_*s*_, was 47 Hz below the *f*_*p*_ and had an intensity, *L*_*s*_, of 70 dB SPL. SFOAE amplitudes were plotted as a function of *L*_*p*_ (5–50 dB SPL in 5-dB steps) in SFOAE I/O function testing to determine the suppression criterion in the testing of SFOAE STCs. The suppression criterion is defined as the SFOAE decrease relative to the total SFOAE [30]. In this study, the suppression criterion was −6 dB, corresponding to an SFOAE amplitude decrease relative to the total SFOAE by a factor of 1/2, because 20log_10_ (1–1/2) = −6 dB. Finally, the SFOAE STCs testing and PTCs testing were conducted separately and then compared. For ease of comparison, the same *f*_*p*_ and *L*_*p*_ were adopted in both tests. The SFOAE STCs *f*_*s*_ was varied from 0.5*f*_*p*_ to 2.5*f*_*p*_ with a resolution of 10 points/octave at a CF of 1,000 Hz and from 0.5*f*_*p*_ to 1.75*f*_*p*_ at CFs of 2,000 and 4,000 Hz, respectively.

### Design of a faster PTC detection algorithm

The methods of Sek *et al.* [24] and Malicka *et al.* [25] formed the basis of the PTC measurements. A simultaneous masking PTC was constructed from different masker intensities at each masker frequency, by fixing the probe frequency and intensity. At each masker tone, the intensity was varied at a rate of 2 dB/s until the subject could not hear the probe tone. The determination of the critical masker intensity was repeated at each masker frequency, and a plot of the masker level at each test frequency yielded the PTC [33].

#### Stimuli

The stimuli comprised the probe and masker tones, delivered by two different speakers. One speaker produced a constant probe tone at a fixed frequency and sound level, whilst the other speaker simultaneously produced a narrowband masking noise with slowly changing center frequency (from low to high: upward sweeps, or from high to low: downward sweeps) and changing sound level as a masker. Each probe cycle was 700 ms, made up of a 200-ms interval and a 500-ms tone, for a total duration of 245 s within 350 cycles. To help the subjects maintain their attention, the probe was pulsed on and off at a fixed rate, with an interval of 200 ms. The 500-ms tone consisted of a 20-ms rise and decay time that reduced the spectral splatter of a rapidly changing tonal intensity. The rise and decay of the envelope of the tone was windowed by a cosine gate function. A 240-s masker was generated 5 s after the probe, to enable the subject to confirm the target signal. The influence of beat detection or an overly wide noise bandwidth can result in a broadened tip of the PTC. Therefore, the bandwidth of the noise masker, at ≤ 90 dB SPL, was 0.2 times the frequency of the probe tone (but always ≤ 320 Hz) [24]. Subjects were instructed to press/release a button when the probe was audible/inaudible. The level of the masking noise was decreased/increased at a fixed rate (2 dB/s). A 245-s downward sweep was made immediately after the 245-s upward sweep to minimize the effects of the narrowband noise sweep. The final PTC was averaged over both the downward and upward sweep procedures.

#### Masker synthesis

The 240-s narrowband noise masker sweep, *S*(t), in the faster PTC consisted of 750 segments, *S*_i_(t), of 640-ms duration. The two adjacent segments were overlapped in time by 50%, as shown in Equation (1).
1

where
2

is the Hanning window and *T* is the period (0.64 s). Each segment, *S*_i_(t), is the narrowband noise with a fixed center frequency. There was a slight difference between the two neighboring segments (*f*_*i* + 1_ is 1.00185 times *f*_*i*_ in the upward sweep), which indicated that the center frequency of the masker changed ±1 octave relative to the probe frequency in 750 segments. For each 640-ms segment the bandwidth was too low to obtain the ideal band-pass filter, so narrowband noise was the inverse fast Fourier transform of a low-pass noise, modulated by multiplying by a sine signal with the same frequency as the center frequency of the narrowband noise.

#### Estimation of tip frequency

The two-point average smoothing method was used to find the trend and estimate the tip frequency of the raw jagged masker intensity curves for upward/downward sweep. The first step was to find the turning points of the raw jagged data, which is hard to quantitatively analyze, and the second step was average smoothing. As the raw data was discrete and the derivative of the two turning points changed sign, the turning points were identified as the non-zero points after the raw data convoluting the filtering operator [1,-2, 1]. Then the smoothed data was set up of the midpoints of the two adjacent turning points. Tip frequency was estimated from the smoothed data. For upward and downward sweeps, each frequency axis was normalized by the tip frequency. Finally, the PTC was the average of both upward and downward sweeps.

### Design of the SFOAE detection method

SFOAE fine structure, I/O function and STC were derived from the recorded SFOAEs with a procedure based on the two-tone suppression method of Brass *et al*. [34, 35]. Those studies used the summation of a four-interval sequence to cancel the probe and suppressor, leaving a residual arising from the nonlinear interactions between the probe and suppressor. In our modified procedure, a single SFOAE detection consisted of stimulation-acquisition, signal detection, data filtering and superposed averaging. At each suppressor tone, the level was varied until the SFOAE was suppressed by the same amount. The determination of the critical suppressor level was repeated at each suppressor frequency, and a plot of the suppressor level at each test frequency yielded the SFOAE STC.

#### Stimuli

**Figure 2:**
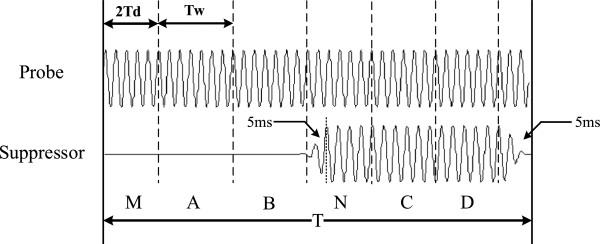
**Stimuli synthesis in a single SFOAE stimulation-acquisition procedure.** The probe signal comprises six sections. The duration of first section is 2*T*d, followed by five sections of duration *T*_w_.

Figure 2 shows the stimuli synthesis for a single SFOAE stimulation-acquisition procedure. To eliminate the effects of system delay and SFOAE latency, section M and N were added to the traditional four-section stimuli paradigm. The stimuli consisted of six sections (except for the last 5 ms). There was one section of 2*T*_*d*_ followed by five sections of *T*_*w*_ (50 ms) in duration. *T*_*d*_ is the system delay from sound-output to signal-input (14.5 ms). The stimuli comprised the probe and suppressor delivered by two different speakers. The probe was a continuous pure tone, with the same polarity in sections A, B, C, D and N. The suppressor was a tone burst, with the rise and decay time of the suppressor envelope windowed by a 5-ms cosine window. Between the rise and decay time of the suppressor tone, the plateau intensity was kept constant. The suppressor in section D was inverted relative to section C.

#### SFOAE detection

Excepting the background noise, the resultant sound in the ear canal consisted of probe artifact, *Rp*, suppressor artifact, *Rs*, the evoked SFOAE signal caused by the probe, *SFE*, the evoked SFOAE caused by the suppressor, *SFEs*, and the remaining SFOAE caused by the probe after suppression, *SFE’*. The collected signals from the ear canal evoked by stimuli in section A to D were stored in four buffers, A to D, respectively. Both buffers A and B contained *Rp* and *SFE*. Buffer C contained *Rp*, *Rs*, *SFEs* and *SFE’*, whilst buffer D contained *Rp*, −*Rs*, −*SFEs* and *SFE’*. The final result, the suppressed SFOAE, is the subtraction of the sound field in sections (A + B) and (C + D) (see Equation 3). It can be seen that the subtraction cancels out the *Rp*, *Rs,* and *SFEs*. If SFOAE can be suppressed completely, whereby *SFE*’ equals zero, then the results of sub-averaging only leave a suppressed SFOAE that equals the SFOAE evoked by the probe tone.
3

Equation 3 describes an operation over raw data in the time domain. The resulting waveform of SFOAE was a signal in the frequency domain after a fast Fourier transform of a suppressed SFOAE. After each stimulation-acquisition process, a zero phase shift high-pass filter (cut-off *f* = 500 Hz) was used to filter low-frequency background noise from the signal. Normally, 64 sub-averages were superposed and averaged after data filtering.

#### SFOAE STCs

A SFOAE STC is a plot of the critical level suppressed the evoked SFOAE to the same criterion as a function of suppressor frequency, at fixed probe frequency and probe level. In our study, the criterion was −6 dB, which means the evoked SFOAE was 50% suppressed. The SFOAE STC took about 30 min to obtain, at a frequency resolution of 10 points/octave.

## Results

### Test results

#### PTCs

**Figure 3:**
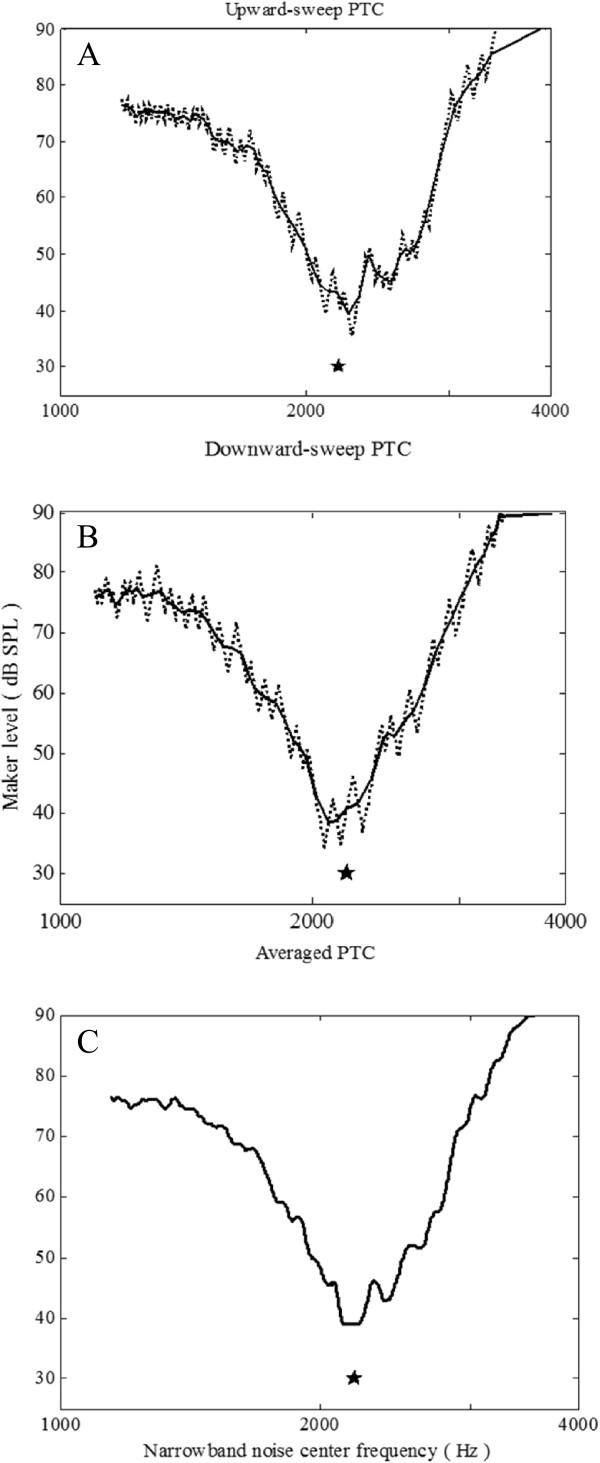
**Example of a fast PTC obtained from one participant with normal hearing. (A)** Upward sweep PTC. **(B)** Downward sweep PTC. **(C)** Averaged PTC. Probe frequency and level are indicated by stars. Dashed jagged lines indicate raw data. Solid lines indicate smoothed data.

Figure 3 shows the faster PTC results for one subject. The results in the faster PTC show the approximate V-shaped curve with a tail, which agrees with the results of Sek *et al.* [24] and Malicka *et al.* [25]. The tips of the upward and downward sweeps trended towards high and low frequencies, respectively, owing to the interference of the sweeping direction. It can be resolved by two-direction averaging (see Figure 3C). The results of subjectively measured PTC may be influenced by non-auditory factors, such as if the subject concentrated on the test and reacted rapidly. It would mean that if the jagged raw data fluctuated more slightly, a better tuning curve could be extracted. However, if the jagged raw data fluctuated too much, then it would be difficult to extract tuning curves accurately.

#### SFOAEs

**Figure 4:**
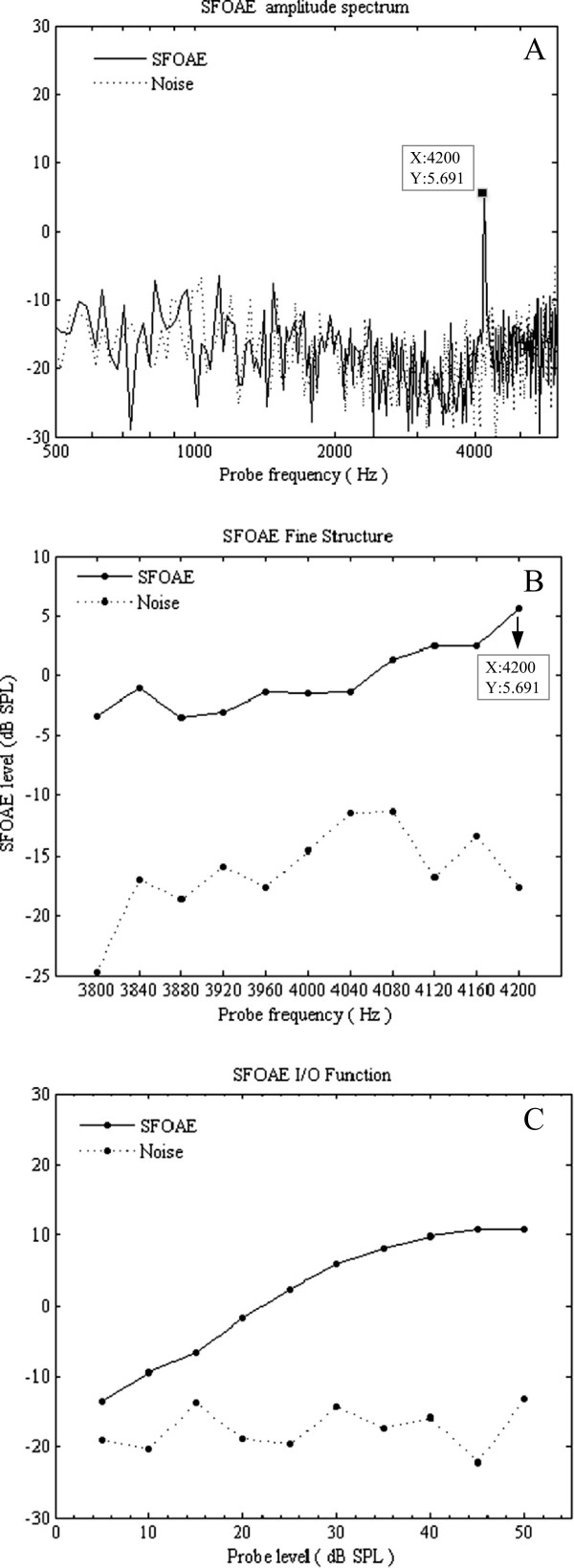
**SFOAE test results for one participant at a CF of 4 kHz. (A)** SFOAE amplitude spectrum. **(B)** SFOAE fine structure. **(C)** SFOAE I/O function. Solid lines indicate SFOAEs. Dotted lines indicate noise. The *L*_*p*_ is 30 dB SPL.

Figure 4 presents an example of the SFOAE test results at a CF of 4 kHz and *L*_*p*_ of 30 dB SPL. The amplitude spectrum contains information in the frequency domain at a *f*_*p*_ of 4,200 Hz which evoked the largest SFOAE in the test of SFOAE fine structure. Extracted SFOAEs had high signal to noise ratios. For this subject, the SFOAE fine structure results showed a best frequency at 4,200 Hz (for a CF of 4 kHz). From 5 to 50 dB SPL, the testing of the SFOAE I/O function shows an increasing function that begins to exhibit a saturation. The results of SFOAE I/O function offered the intensity of the evoked SFOAEs at the probe frequency and probe level, which can be used to choose the appropriate criterion in the test of SFOAE STCs.

### Comparison between SFOAE STCs and PTCs

**Figure 5:**
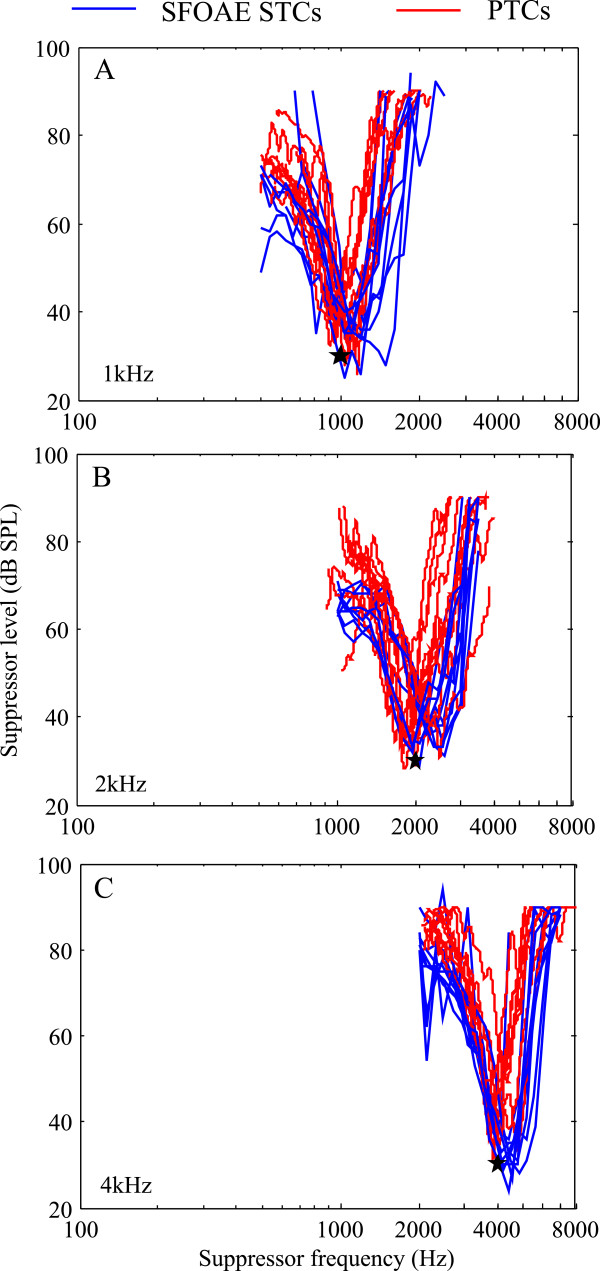
**Comparison between SFOAE STCs (blue) and PTCs (red) for all subjects. (A)** CF = 1 kHz. **(B)** CF = 2 kHz. **(C)** CF = 4 kHz. The probe frequency and level are indicated by black stars.

Using a logarithmic frequency axis and decibel intensity axis, we compared the SFOAE STCs and PTCs for all subjects at a suppression criterion of −6 dB (Figure 5). The results show that SFOAE STCs shift higher relative to *f*_*p*_, whilst PTCs shift similarly to *f*_p_. The overall shapes of the SFOAE STCs and PTCs of all subjects showed similar trends, except for a shift of the tip, which suggests that a potential use of SFOAE STCs may be as an objective measure of FS, equivalent to PTCs.

#### Q_10_ values

**Figure 6:**
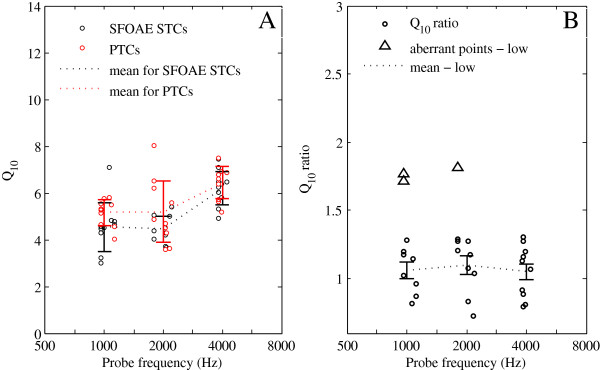
**Q**_**10**_ **values and Q**_**10**_ **ratio for SFOAE STCs and PTCs. (A)** Q_10_ values (circles) and mean Q_10_ values (dotted lines) for SFOAE STCs (black) and PTCs (red) as a function of probe frequency. **(B)** Q_10_ ratio (black circles) and mean Q_10_ ratio (dotted line) as a function of probe frequency. Aberrant points indicated by triangles. *Error bars* denoted as ± 1 SE.

The Q_10_ value was calculated as the ratio between the tip frequency of the tuning curve and the bandwidth of the tuning curve 10 dB above the tip. Q_10_ values of SFOAE STCs and PTCs for all subjects are shown in Table 1. Mean Q_10_ values increased for both SFOAE STCs and PTCs as a function of CFs (Figure 6A)*.* For both SFOAE STCs and PTCs, Q_10_ values at a CF of 1 kHz are closer to the values at a CF of 2 kHz, but Q_10_ values at a CF of 4 kHz are much larger. The paired *t* test indicates that Q_10_ values of SFOAE STCs and PTCs are significantly different (*M*_*D*_ = −.5, *SD* = 1.20, *t* = −2.419, *p* = .022). Q_10_ values of PTCs are larger than SFOAE STCs at all CFs.

**Table 1:** **Q**_**10**_ **values of SFOAE STCs and PTCs for all subjects**

*Subject No.*	*CF = 1000 Hz*			*CF = 2000 Hz*			*CF = 4000 Hz*		
	*f*_*p*_ *(Hz)*	*Q*_*10*_ *_STC*	*Q*_*10*_ *_PTC*	*f*_*p*_ *(Hz)*	*Q*_*10*_ *_STC*	*Q*_*10*_ *_PTC*	*f*_*p*_ *(Hz)*	*Q*_*10*_ *_STC*	*Q*_*10*_ *_PTC*
1	1120	4.76	4.57	1800	4.40	8.01	4200	6.45	6.88
2	960	4.29	5.14	2080	3.66	4.29	3960	6.36	5.16
3	1120	4.65	4.03	2200	5.38	5.58	3840	7.43	6.60
4	1080	4.81	5.51	2040	3.69	4.71	3840	4.90	6.40
5	960	3.22	5.52	1800	5.06	6.51	3840	5.31	6.76
6	960	4.55	4.66	2040	4.20	4.53	3840	7.11	5.64
7	1060	7.11	5.79	2160	5.00	3.64	3840	6.45	7.49
8	960	4.46	5.25	1800	4.86	6.21	3800	6.01	6.80
9	960	3.01	5.33	2040	4.30	3.57	3800	6.26	5.72
10	1000	4.50	5.76	1800	4.04	4.86	3960	5.79	6.92
Mean		4.53	5.16		4.46	5.19		6.21	6.44
Standard deviation		1.0994	0.5705		0.5919	1.3921		0.7589	0.7117

Q_10__STC and Q_10__PTC represents the Q_10_ value of SFOAE STC and PTC, respectively. *f*_*p*_ represents the best frequency of the probe in SFOAE Fine Structure (i.e., frequency that can evoke the largest SFOAE).

#### Q_10_ ratio

To explore the relationship between the frequency selectivity of SFOAE STCs and PTCs, we calculated Q_10_ ratios (Q_10_ values of the PTCs divided by the Q_10_ values of the SFOAE STCs).The mean values of the Q_10_ ratios remain relatively constant across CFs at low probe levels (*F*_2,24_ = .15, *p* = .858), except for the aberrant points indicated by triangles (Figure 6B). At all CFs, the mean Q_10_ ratios are approximately 1 (*M* = 1.059, *SD* = .168 at a CF of 1 kHz; *M* = 1.099, *SD* = .202 at a CF of 2 kHz; *M* = 1.054, *SD* = .190 at a CF of 4 kHz), which suggests that the frequency selectivity of SFOAE STCs is similar to that of PTCs.

#### Offset ratio

The horizontal and vertical offset ratios are the percentages of the difference of frequency, *f*_tip_, and tip level, *L*_tip_, relative to *f*_p_ and *L*_p_, as shown in Equations (4) and (5) respectively.
45

**Figure 7:**
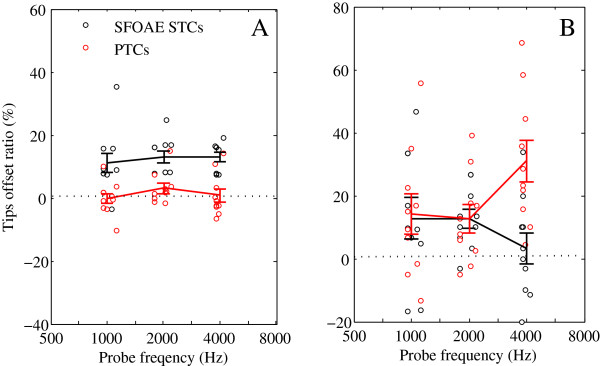
**Offset ratios for SFOAE STCs and PTCs as a function of** ***f***_**p**_ **.** Horizontal **(A)** and vertical **(B)** offset ratio for SFOAE STCs (black) and PTCs (red) at different CFs. Offset ratios, circles; mean offset ratios, solid lines.

The differences between tip frequencies and levels were calculated as *f*_tip_-*f*_p_ and *L*_tip_-*L*_p_, respectively. The horizontal and vertical offsets could reflect the features of the site with the sharpest tuning on the basilar membrane. At different CFs, horizontal and vertical offset ratios are not significantly different for either SFOAE STCs or PTCs (SFOAE STCs: *F*_2,27_ = .22, *p* = .806 for horizontal offset ratio, *F*_2,27_ = 1.15, *p* = .331 for vertical offset ratio; PTCs: *F*_2,27_ = .83, *p* = .449 for horizontal offset ratio, *F*_2,27_ = 3.02, *p* = .065 for vertical offset ratio). For horizontal offset ratios, SFOAE STCs are significantly larger than PTCs (*M*_*D*_ = 11.1, *SD* = 8.32, *t* = 7.335, two-tailed *t* test *p* < .001), and PTCs are mostly scattered around 0 (Figure 7A). SFOAE STCs shift higher relative to *f*_*p*_, but the PTCs shift always coincided with *f*_p_. For vertical offset ratios, the SFOAE STCs and PTCs are both shifted higher relative to *L*_*p*_ (*M*_*D*_ = −9.7, *SD* = 26.86, *t* = −1.969, two-tailed *t* test *p* = .059). SFOAE STCs are similar to PTCs at CFs of 1 and 2 kHz, but much smaller than PTCs at a CF of 4 kHz (Figure 7B).

## Discussion

### Effectiveness

The SFOAE STC measurement took 30 min at a frequency resolution of 10 points/octave, and 15 min at a frequency resolution of 5 points/octave. The fast PTC took **~**8 min to obtain, with the masker frequency changing ±1 octave relative to the probe frequency. The SFOAE STC was therefore more time-consuming. However, at a CF of 4,000 Hz, the tip masker level of the PTC was much larger than the SFOAE STC (Figure 7B). This indicates that the subject will feel more uncomfortable during the detection of PTCs at higher masker frequency. The interpretation of PTCs was influenced by non-auditory factors whilst the SFOAE determination was not. Therefore, SFOAE STCs have more potential for auditory function assessment when compared with PTCs.

### Q_10_ value

The result indicates that the mean values of Q_10_ ratios of PTCs to SFOAE STCs were around 1, and remained relatively constant across CFs at low probe levels. However, Q_10_ values of PTCs were still a little larger than SFOAE STCs at all CFs which seems that PTCs are more sharply tuned than SFOAE STCs. It may be reasonable because PTCs reflect the FS characteristic of the auditory propagation pathway as a subjective measurement, but SFOAE STCs reflect the FS of auditory periphery as an objective measurement.

### Tip frequency offset

The tip frequency of SFOAE STCs shifted higher than *f*_*p*_, but the shift of PTCs always coincided with *f*_*p*_ (Figure 7A). Our findings are similar to those of other SFOAE studies [30, 32]. The mean tip frequency offset of SFOAE STCs was 12.36% (*M* = 11.13%, *SD* = 10.008 at a CF of 1 kHz; *M* = 12.97%, *SD* = 5.920 at a CF of 2 kHz; *M* = 12.99%, *SD* = 4.744 at a CF of 4 kHz), which is a shift of 1.12*f*_*p*_. This demonstrates that the more sharply tuned frequencies were located basal to the characteristic place of the probe frequency.

## Conclusion

We designed an assessment method of human auditory FS using the detection of both PTCs and SFOAE STCs. The effectiveness of the objective SFOAE STCs method to the subjective PTCs method was compared in 10 individuals with normal hearing at low probe levels. Our results showed that estimates of FS provided by the SFOAE STCs were similar to those provided by behavioral measures of PTCs, suggesting that SFOAE STCs have the potential to assess frequency selectivity in a noninvasive, objective and effective way.
